# Multidirectional Effects of Red Clover (*Trifolium pratense* L.) in Support of Menopause Therapy

**DOI:** 10.3390/molecules28135178

**Published:** 2023-07-03

**Authors:** Anna Gościniak, Piotr Szulc, Waldemar Zielewicz, Jarosław Walkowiak, Judyta Cielecka-Piontek

**Affiliations:** 1 Department of Pharmacognosy and Biomaterials, Poznan University of Medical Sciences, Rokietnicka 3, 60-806 Poznan, Poland; anna.gosciniak@student.ump.edu.pl (A.G.); jpiontek@ump.edu.pl (J.C.-P.); 2 Department of Agronomy, Poznan University of Life Sciences, Dojazd 11, 60-632 Poznan, Poland; piotr.szulc@up.poznan.pl; 3 Department of Grassland and Natural Landscape Sciences, Poznan University of Life Sciences, Dojazd 11, 60-632 Poznan, Poland; waldemar.zielewicz@up.poznan.pl; 4 Department of Pediatric Gastroenterology and Metabolic Diseases, Poznan University of Medical Sciences, Szpitalna Str. 27/33, 60-572 Poznan, Poland

**Keywords:** isoflavones, red clover, varieties, pleiotropic, menopause

## Abstract

Red clover is a raw material of interest primarily due to its isoflavone content. However, other groups of compounds may affect the pleiotropic biological effects of this raw material. It is used to alleviate menopausal symptoms, but the fact that there are many varieties of this plant that can be grown makes it necessary to compare the biological activity and phytochemical composition of this plant. Also of interest are the differences between the leaves and flowers of the plant. The aim of this study was to evaluate the properties of the leaves and flowers of six clover varieties—‘Tenia’, ‘Atlantis’, ‘Milena’, ‘Magellan’, ‘Lemmon’ and ‘Lucrum’—with respect to their ability to inhibit α-glucosidase, lipase, collagenase and antioxidant activity. Therefore, the contents of polyphenols and the four main isoflavones—genistein, daidzein, biochanin and formononetin—were assessed. The study was complemented by testing for permeability through a model membrane system (PAMPA). Principal component analysis (PCA) identified a relationship between activity and the content of active compounds. It was concluded that antioxidant activity, inhibition of glucosidase, collagenase and lipase are not correlated with isoflavone content. A higher content of total polyphenols (TPC) was determined in the flowers of red clover while a higher content of isoflavones was determined in the leaves of almost every variety. The exception is the ‘Lemmon’ variety, characterized by high isoflavone content and high activity in the tests conducted.

## 1. Introduction

**Figure 1:**
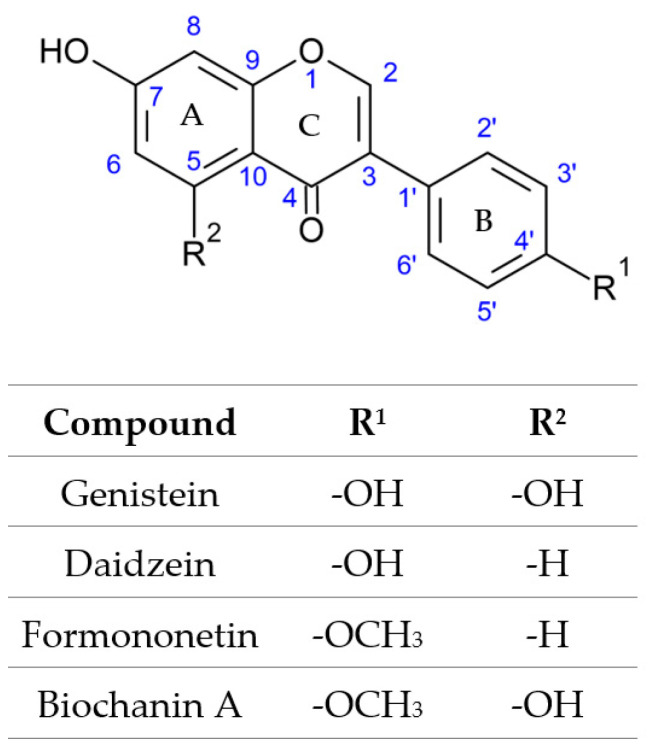
Chemical structures of isoflavones—genistein, daidzein, formononetin, biochanin.

The search for natural ways to alleviate the effects of menopause is an interesting solution to improve women’s comfort. The most well-known use of substances of natural origin in menopause is the use of isoflavones. Some of the most widely distributed isoflavones are genistein, daidzein, formononetin and biochanin A (Figure 1). Their most common source is soybeans, but other raw materials rich in these chemicals, such as clovers, are being intensively studied [1]. Red clover is the raw material from which these isoflavones can be effectively isolated [2]. The use of preparations rich in isoflavones genistein and daidzein is beneficial for mood disorders, sedation and hot flashes, among others [3,4,5]. Although scientific interest has mainly focused on soy as the most popular commodity for menopause, there is also scientific evidence of the health-promoting effects of *Trifolium pratense* L., red clover. A meta-analysis by Ghazanfarpour et al. [6] found that clover can alleviate menopausal symptoms such as hot flashes and vaginal dryness. The effect of red clover on the lipid profile of postmenopausal and premenopausal women is interesting—it turns out that red clover may positively affect the lipid profile, as inferred by Luis et al. [7] in a meta-analysis of available scientific reports. There is also evidence of red clover being effective in lowering blood glucose and having beneficial effects on the prevention of cardiovascular disease [7,8]. In an in vitro model, its anti-inflammatory and antioxidant effects were documented in lipopolysaccharide-stimulated macrophages [9,10].

However, only one scientific study has examined the difference in activity between different varieties of *T. pratense* and documented significant differences [11]. Petrauskas et al. studied the genetic structure of wild red clover populations and local varieties in Lithuania, which is located in a nemoral environmental zone. They found high genetic diversity within red clover populations and local cultivars [12]. Moreover, studies of other raw materials have shown that differences between varieties can have important implications for biological activity and chemical composition [13].

Additionally, research to date has mainly focused on clover leaves as a raw material rich in isoflavones [14,15]. Only a few publications have also characterized the multidirectional activity of this raw material. Kazlauskaite et al. [16] tested how the extraction efficiency is affected by the addition of cyclodextrin and also evaluated the antioxidant potential, which is very important in the prevention of cancer and cardiovascular disease as well as affecting the damage of cells, such as pancreatic cells, which affect the development of diabetes. Gligor et al. [17] investigated the effect of the extraction method used on the activity and content of the extracts tested. The extracts showed a high potential against Gram-negative bacteria and induced a modest antioxidant effect on the experimental inflammation model in Wistar rats. A review of research by Mohsen et al. [18] revealed that recent studies have focused on the effects of red clover exhibiting antioxidant and anticancer effects. In addition, they exert beneficial effects on cardiovascular function and improve menopausal symptoms. In addition, these compounds can regulate blood glucose levels and lipid markers. The effects of this raw material have also been studied on various tissues, including the endometrium, breast, skin and reproductive system.

In view of the reports above on the normalization of the lipid profile after the consumption of clover preparations, it is interesting to note the possibility of inhibiting enzymes responsible for the breakdown of fats. To date, in silico studies have confirmed the potential of isoflavones to inhibit lipase [19]. Tundis et al. [20] demonstrated that clover flower extract inhibits α-glucosidase, α-amylase and also lipase. However, it is highly interesting to compare the activity and content of active compounds in the leaves and flowers of clover of different varieties.

The aim of the study was to evaluate the content of isoflavones in the leaves and flowers of red clover of six varieties. The content of total polyphenols was assessed, and the activity of the prepared extracts was compared using two tests of antioxidant activity and enzyme inhibition—glucosidase, lipase and collagenase. In addition, permeability through an artificial membrane system simulating three different conditions—the blood–brain barrier, the gastrointestinal tract and the skin—was evaluated.

## 2. Results and Discussion

### 2.1. HPLC Analysis

**Figure 2:**
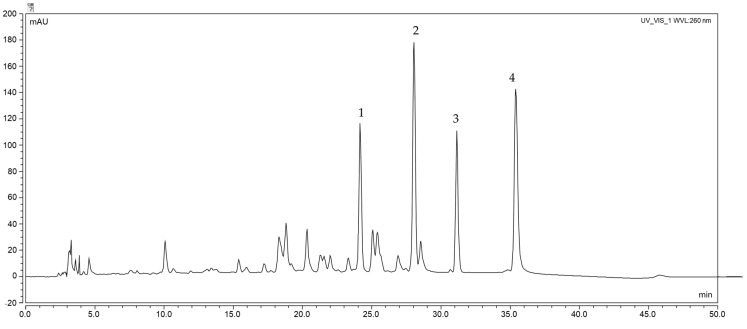
High-performance liquid chromatography (HPLC) chromatogram profiling of *Trifolium pratense* L. extracts (variety ‘Tenia’). 1—genistein, 2—daidzein, 3—formononetin, 4—biochanin A.

**Figure 3:**
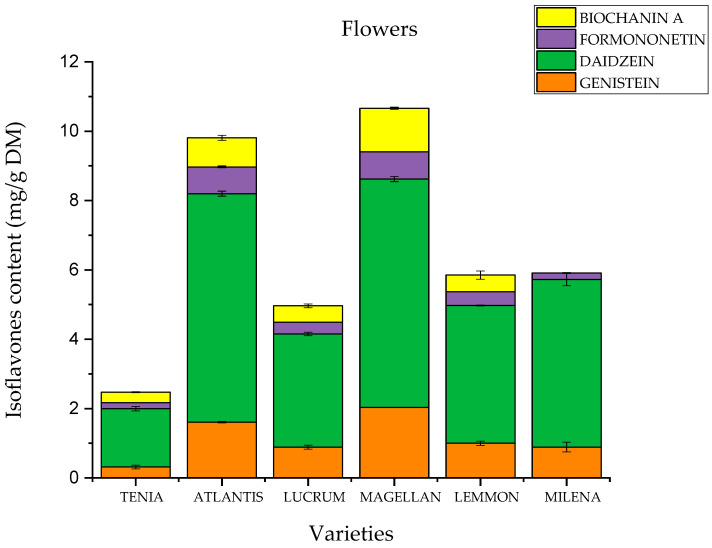
Content of active compounds in the flowers of *Trifolium pratense* L. extracts.

**Figure 4:**
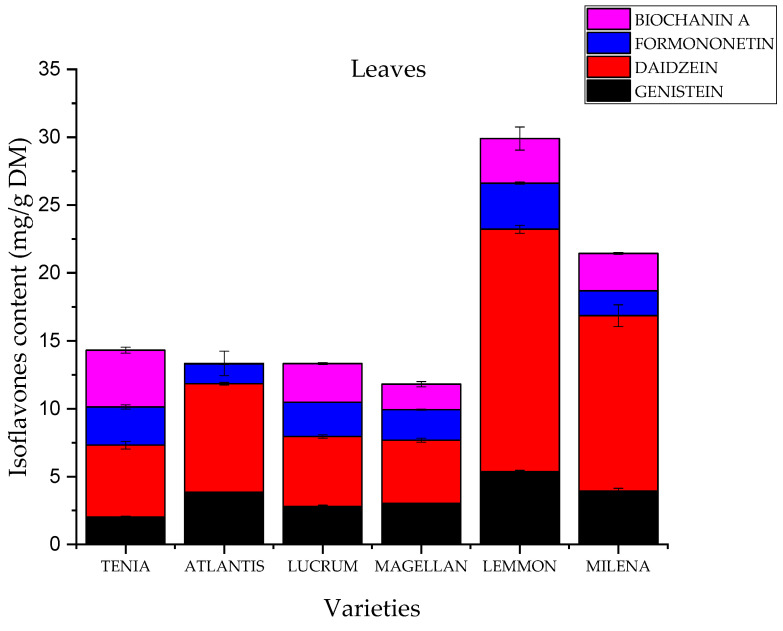
Content of active compounds in the leaves of *Trifolium pratense* L. extracts.

The composition and content of active compounds were assessed by the HPLC method. The compounds assessed were the isoflavones genistein, daidzein, formononetin and biochanin A (Figure 2). They are secondary plant compounds with structural similarity to the endogenous steroid hormone 17β-estradiol and are called phytoestrogens [21]. Figure 3 and Figure 4 show the results of the determination. Red clover leaves are characterized by a higher content of isoflavones. The total content of the four isoflavones varied between 29.904 mg/g in ‘Lemmon’ leaves and 6.246 mg/g in ‘Magellan’ leaves. Flowers show a lower sum of isoflavones between 3.260 mg/g for the variety ‘Milena’ and 0.733 for the variety ‘Tenia’. A higher content of isoflavone in leaves than in flowers was also shown by Lemežienė et al. [22] in their study; however, the proportion of genistein and daidzein, the unmethylated forms, was much lower than that of the methylated forms biochanin A and formononetin. In our study, it was shown that the content of unmethylated forms in the varieties studied is similar to that of methylated forms. Soya is considered to be the raw material that contains higher amounts of genistein and daidzein, but our study showed that their content in red clover could also be high. The polyphenol content depends on many factors, such as the variety of the raw material, the place of cultivation, the soil and the climatic conditions [23,24,25]. Furthermore, the content of formononetin and genistein also remains high. The differences may be due to the preparation of the raw material. In our study, we used fresh raw material, in contrast to Lemežienė et al. [22], who extracted dried raw material. The isoflavone content was higher in the leaves, which is in line with the study conducted by Saviranta et al. [26]. As in the Tsao et al. [11] study, mainly aglycone content was detected. This may be influenced by sample preparation. Research has indicated that despite their stability, isoflavone glycosides can undergo hydrolysis due to the activity of the enzyme β-glucosidase. This enzyme can be triggered when tissues are subjected to damage. The conditions of sample preparation—freezing and subsequent freeze-drying—were similar to those in the study by Tsao et al., which may further prove this relationship. In contrast, a different conclusion was reached by Swinny et al. [27], who demonstrated that post-harvest freeze-drying inhibited the conversion of glycosides to aglycones, while vacuum drying allowed maximum conversion of glycosides to their corresponding aglycones. In the human body, glycosides can be converted into their corresponding aglycones by enzymes present in the gut, and then also into active compounds such as equol in the case of genistein formed in the presence of the bacterium *Slackia isoflavoniconvertens* [28].

The fact that different varieties had different levels of isoflavones suggests a genetic influence on isoflavone biosynthesis; thus, isoflavone concentrations can potentially be raised to higher levels through breeding, which in turn promotes the extraction of raw material with the most favorable properties. It can be seen that the flowers of tetraploid varieties have a higher isoflavone content.

### 2.2. Determination of Total Phenolic Content (TPC)

The total polyphenols in the red clover varieties are shown in Table 1. The flowers have a higher polyphenol content. There are also statistically significant differences between the varieties, which are also presented in Table 1. A variety worth highlighting is the ‘Lemmon’ variety, in which the leaves are characterized by their high polyphenol content (40.584 ± 0.976 mg/g) and the value is similar to the flowers in this variety (43.401 ± 1.317 mg/mL). The results are similar to those of other researchers; Esmaeili et al. [29] showed that the polyphenol content of the aboveground part of the plant was 46.88 ± 1.07 mg GAE/g DW. Considering that the leaves of this variety, as mentioned earlier, have a high content of isoflavones, this variety is promising for the properties it possesses. Of the flowers of other varieties, the ‘Lucrum’ variety has the highest content of polyphenols (56.178 ± 1.102). It should be noted, however, that despite the higher values for total polyphenols, clover flowers had a lower content of isoflavones. It should therefore be suspected that isoflavones are not the main polyphenolic compounds. Leaves that showed a lower content of total polyphenols have up to 10 times higher isoflavone content. However, when we dissect the polyphenol content of the different varieties, there is a correlation between polyphenol content and isoflavone content (*p* < 0.05). Kicel et al. [30] showed that the flowers are characterized by a higher content of one of the polyphenol groups, phenolic acids. It can therefore be concluded that the content of the different groups in clover flowers and leaves differs significantly, and because polyphenol content is linked to biological activity, this can affect a variety’s performance profile. To date, few studies have analyzed the full polyphenolic profile of red clover. However, the presence of other phenolic compounds is highly significant for activity, as illustrated by our study. Extracts with a high content of isoflavones do not always show the strongest activity and have also been found to contain other polyphenols. Vlaisavljević et al. [31] identified in the extract, in addition to the main isoflavones, compounds such as p-hydroxybenzoic and caffeic acid, kaempferol 3-*O*-glucoside, quercetin 3-*O*-glucoside, hyperoside and aesculetin, as well as glycosides kaempferol 3-*O*-glucoside, quercetin 3-*O*-glucoside and apigenin 7-*O*-glucoside. Investigating the content of other polyphenols is an interesting direction for future research.

Different polyphenol content in particular parts of the raw material is a characteristic of the species. In the case of extracts prepared from the leaves and flowers of dandelion (*Taraxacum officinale* F. H. Wigg.), higher contents were harbored by the leaves than by the flowers [32]. However, in the case of wild garlic (*Allium ursinum* L.), the polyphenol content was higher in the flowers [33].

### 2.3. Antioxidant Activity

Antioxidant activity plays an important role in counteracting pathogenic processes such as cardiovascular disease, chronic obstructive pulmonary disease, chronic kidney disease, neurodegenerative diseases, and cancer [34]. Red clover is known as a resource for use in menopausal symptoms. The antioxidant effect shows a multidirectional action in diseases whose risk increases with age such as cardiovascular and neurodegenerative disease, an additional benefit. Potential antioxidant activity was tested using DPPH and CUPRAC. These methods concern different ways of testing antioxidant potential: free radical scavenging and metal reduction. Red clover flowers and leaves exhibit antioxidant activity. The clover flower extract shows stronger antioxidant activity than the leaves, which is in line with the conclusions from the polyphenol content. The exception is the variety ‘Lemmon’, in which it was the leaves that showed very strong antioxidant activity, the same as flowers. The post hoc test showed statistically significant differences between the varieties (Table 2). For both methods, the ‘Lemmon’ leaf variety showed the strongest antioxidant activity, 0.683 ± 0.037 mL and 2.583 ± 0.089 mg/mL, respectively. Among the flowers, the ‘Lucrum’ variety had the strongest activity, 0.644 ± 0.004 and 1.393 ± 0.096, respectively, for the two methods. The significance of the statistical differences between the varieties is also shown in Table 1. It has been proven repeatedly that a higher content of polyphenols is associated with higher antioxidant and biological activity [35,36,37]. We can also note a correlation between isoflavone content and antioxidant activity—varieties with higher isoflavone content show stronger antioxidant activity. This effect is not observed in the case of flowers. This can be explained by the fact that in leaves, the content of isoflavones is only a fraction of the polyphenols present, while in flowers, it is a much higher percentage. Compared to soybeans which are also used as a raw material rich in isoflavones, red clover appears to be a better antioxidant. Samruan et al. [38] showed that the IC_50_ values for the aqueous and alcoholic dry extracts of soybeans are 21.091 ± 0.558 and 41.294 ± 3.184 mg/mL, respectively. Consequently, the results obtained in this study show the significantly stronger antioxidant potential of red clover. Kazlauskaite et al. showed that cavoylmalic acid present in clover extract gave the highest antioxidant response compared to other compounds in the extract. Moreover, a rather high response of hyperoside was also detected.

### 2.4. α-Glucosidase Inhibition

One of the main mechanisms on which the antidiabetic action of plant raw materials is based is the inhibition of enzymes responsible for the breakdown of complex sugars. A-glucosidase continues the process started by α-amylase by further breaking down into glucose which enters the circulation. The effect of this is that there is no sudden rise in blood glucose after carbohydrate consumption. This is particularly important in view of the fact that menopause is also a time when insulin resistance appears, and special attention should be paid to sugar metabolism. On the basis of the study, it can be concluded that both red clover leaves and flowers show the potential to inhibit α-glucosidase (Table 3). However, red clover flowers show a stronger potential for α-glucosidase inhibition. The leaf extract of the ‘Lemmon’ variety showed the strongest antidiabetic activity, but this was an exception to the observed rule where it was the flowers that showed stronger inhibitory activity. Once again, the ‘Lemmon’ variety shows the most beneficial properties because it inhibits α-glucosidase strongly and has a high isoflavone content. In leaves, we observe a correlation showing a dependence between the sum of polyphenols and antidiabetic activity. In the work of Masuda et al. [9], formononetin, an α-glucosidase inhibitor, was suspected to be the main functional molecule responsible for the antidiabetic effect. Masuda et al. [7] suggest that a daily intake of 1.91 g of red clover extract (containing 8 mg formononetin and 1.8 mg biochanin A) can lower blood glucose levels, especially in people aged ≤ 50 years. Most studies link antidiabetic activity to the presence of isoflavones. However, the results of our study are not consistent with this conjecture. Leaves containing a higher formononetin content do not inhibit this enzyme more strongly. However, in such a complex matrix as plant raw materials, the more important is the so-called entourage effect—the phenomenon best studied for hemp raw materials involving higher activity of all components present in the raw material than single effects. It is not excluded that it is not the isoflavones that are responsible for this activity, but the synergistic action of the ingredients may be more important. The potential for α-glucosidase inhibition by red clover flower extract was also confirmed by Tundis et al. [20]. However, it should be mentioned that, despite its activity on α-glucosidase, the raw material should be characterized as weakly active. Acarbose, a drug used in diabetes, inhibits 50% of the activity at a concentration of 2.871 ± 0.044 mg/mL, while raw materials such as aloe vera gel show an IC_50_ value of 0.598 ± 0.013 µg/mL. The potential of this raw material has also been confirmed by animal studies [39] and clinical trials. Oza et al. [40] reported results from a rat study showing that an aqueous extract of *Trifolium pratense* played a beneficial role in improving insulin sensitivity and SIRT1 expression. It is not excluded that isoflavones are responsible for the antidiabetic activity of clover extract, but we suspect that other mechanisms are responsible for this effect, e.g., through the activation of hepatic PPARα/γ and inhibition of hepatic fatty acid synthase, as suggested by Qiu et al. [41].

### 2.5. Collagenase Inhibition

Loss of skin firmness and a change in the appearance of the skin is also a problem during menopause. Plant-based raw materials can improve the appearance of the skin during this period. The increment in collagenase activity also affects the condition of the gums. The mechanisms responsible for this process are decreased collagen and glycosaminoglycan synthesis in periodontal tissue fibroblasts and increased collagenase activity in the tissues [42]. The inhibitory activity of collagenases can be used, among other things, in anti-aging and anti-inflammatory preparations and also in wound treatment. The ability to inhibit collagenase was assessed to evaluate the functional activities of clover flower and leaf extracts (Table 4). Studies have shown that both the flowers and leaves of red clover show the potential to inhibit collagenase. ‘Lemmon’ leaves and ‘Lucrum’ flowers showed the highest activity, with 89.381 ± 4.780 and 89.174 ± 4.206% inhibition, respectively. However, it should be noted that this effect is moderate compared to the reference substrate, epigallocatechin gallate. Again, we can observe that it is the flowers that are more active. In this case, too, the leaves of the ‘Lemmon’ variety show beneficial properties because, with their high isoflavone content, they exhibit strong inhibition. Research again links the beneficial properties of collagenase inhibition to the presence of isoflavones. Alqodri et al. [43] confirmed the inhibitory potential on collagenase for daidzein and Geeta et al. [44] for genistein.

Beneficial properties have been demonstrated with both internal and topical applications. In a study conducted by Manzoureh et al. [45], it was reported that increased collagen levels in wound tissue contribute to the reduction in wound area and improvement in wound contraction. The researchers observed the effects of *Trifolium pratense* (red clover) extract on wound contraction by assessing histological parameters, particularly re-epithelialization. An ointment was used in the study. Circosta et al. [46] studied the effect of a clover extract containing 11% isoflavones on rats after ovarian excision. They showed that the skin of rats after ovarian excision, but after treatment with the extract, appeared well organized with a normal epidermis of uniform thickness and regular keratinization, and blood vessels, collagen and elastic fibers were well developed. The amount of collagen increased significantly in the treated group compared to the control group. The results for biochanin A are also interesting. It appears that treatment with biochanin A inhibited inflammation, regulated the unbalanced oxidative stress response and improved alveolar bone loss in periodontitis [47]. Sin et al. [48] showed that flavonols were more potent inhibitors than flavones/isoflavones. They suggested that the presence of a C-3-hydroxyl group in the flavonol compounds is crucial for their enhanced inhibitory effects. Their study specifically highlights the comparison between apigenin and kaempferol, as well as luteolin and quercetin. This is also confirmed by the results of our study. Despite the higher content of isoflavones in the leaves, it was the flowers, richer in total polyphenols, that showed stronger activity to inhibit collagenase. We may suspect that the synergistic effect of the present compounds may be the key or that other compounds are responsible for the collagenase inhibitory action.

### 2.6. Lipase Inhibition

The menopausal period is associated with an increased risk of weight gain and the associated increased risk of cardiovascular disease [49]. While the priority in maintaining a healthy weight should be a healthy lifestyle and exercise, it appears to be supported by the use of natural compounds to help maintain a healthy weight. Lipase is the enzyme responsible for breaking down fats and allowing them to be absorbed. Lipase inhibitors, by binding to the active part of lipase in the stomach and small intestine, inhibit the catalytic activity and thus reduce the digestion of lipids. Diminished hydrolysis results in decreased absorption of lipids from food and the accumulation of adipose tissue and allows obesity to be controlled. Previous studies have shown that extracts from plant raw materials may have inhibitory activity on lipase and could potentially be used as supportive therapy [50,51,52].

Among the varieties tested, the leaves of the ‘Lemon’ variety showed the strongest activity, while for the flowers, it was the ‘Lucrum’ variety (Table 5). Once again, the flowers showed stronger activity, the exception being the variety ‘Lucrum’, whose leaves showed the strongest activity of all the extracts tested. It can be seen that, again, the stronger enzyme inhibition is related to the higher polyphenol content. To date, the effect of red clover on lipase activity has not been studied. A study by Martinez-Gonzalez et al. [53] found that polyphenolic compounds showed mixed inhibition of pancreatic lipase, with quercetin being the strongest inhibitor tested, with an IC_50_ similar to orlistat. Quercetin is also found in red clover, according to the study by Tava et al. [54], which leads us to believe that in this case, this polyphenol, among others, is involved in lipase inhibition. However, when comparing the results with orlistat, a synthetic substance used as a lipase inhibitor to treat obesity, the results obtained are more than 100 times weaker. No correlation was found between lipase inhibition and isoflavone content. What is significant, however, is the correlation between the sum of polyphenols and the inhibitory activity of the enzyme. Again, it is possible that the synergistic effect is responsible for lipase inhibition or it is the presence of one of the polyphenols whose presence is not tested in this study. However, Guo et al. [55] examined the impact of daidzein on body weight, adipose tissue and lipid levels in obese mice that were fed a high-fat diet. The researchers discovered that daidzein supplementation resulted in a reduction in body weight and white adipose tissue weight in the obese mice. Additionally, daidzein was found to ameliorate the hyperlipidemia caused by the high-fat diet.

Other mechanisms should also be considered. Isoflavones potentially alleviate hyperglycemia and dyslipidemia by activating peroxisome-proliferator-activated receptors (PPARs), which are nuclear receptors involved in cellular lipid balance and insulin function [41].

### 2.7. Parallel Artificial Membrane Permeability Assay (PAMPA)

**Figure 5:**
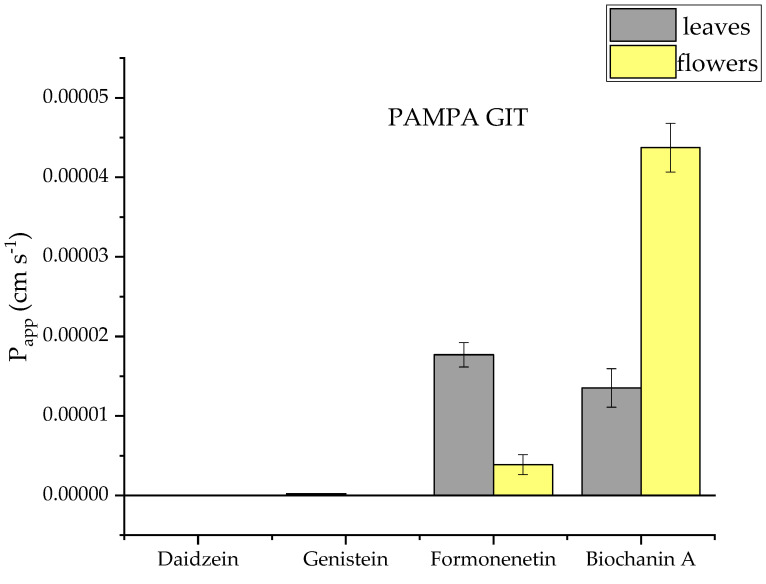
Apparent permeability coefficient (P_app_) of isoflavones from red clover (*Trifolium pratense* L.) using the parallel artificial membrane permeability assay simulating gastrointestinal barrier (PAMPA-GIT).

**Figure 6:**
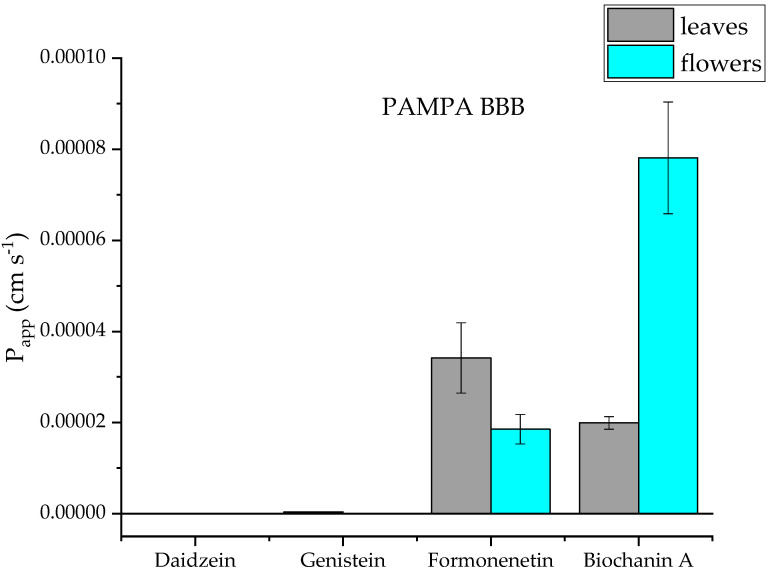
Apparent permeability coefficient (P_app_) of isoflavones from red clover (*Trifolium pratense* L.) using the parallel artificial membrane permeability assay simulating the blood–brain barrier (PAMPA-BBB).

**Figure 7:**
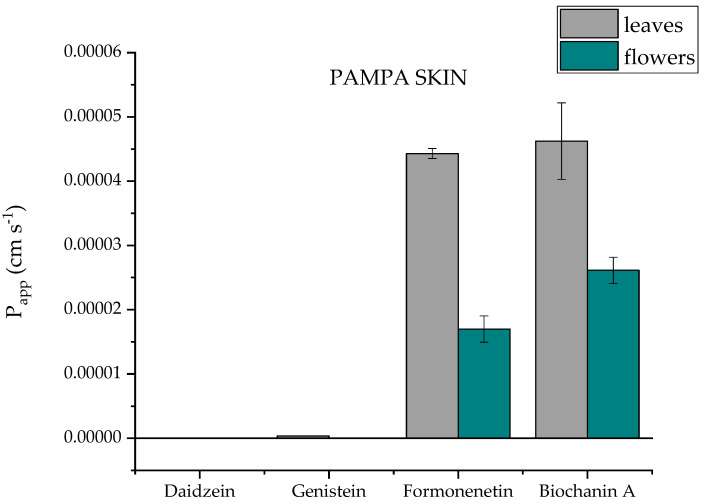
Apparent permeability coefficient (P_app_) of isoflavones from red clover (*Trifolium pratense* L.) using the parallel artificial membrane permeability assay simulating skin (PAMPA-SKIN).

The permeability of genistein, daidzein, formononetin and biochanin A was assessed by a simple method using artificial membranes simulating gastrointestinal transport, the blood–brain barrier and the skin. The parallel artificial membrane permeability assay (PAMPA) is a simple method that assesses passive permeability. On the basis of the study, formononetin and biochanin A were shown to have very good permeability, P_app_ > 1 × 10^−6^ cm/s. Genistein and daidzein did not show such permeability (Figure 5, Figure 6 and Figure 7). The difference in permeability of these isoflavones is probably due to their chemical structure and is connected to the differences in solubility.

Membrane permeability—and therefore, bioavailability—is greater for substances with greater lipophilicity due to the presence of methyl groups present in formononetin and biochanin A [56]. Papaj et al. [57] also showed that the permeability of genistein is low in the PAPMA assay in contrast to derivatives with higher lipophilicity. Luo et al. [58] showed in a rat study that formononetin is mainly absorbed by passive transport and has a bioavailability of 21.8%. Gampe et al. [59] also confirmed that formononetin crosses the blood–brain barrier. However, low permeability in a passive permeability test does not determine the lack of uptake of these compounds. The gut microbiota is also involved in the absorption of isoflavones. Daidzein is converted into dihydrodaidzein, O-desmethylangolensin and equol (7-hydroxy-3-(49-hydroxyphenyl)-chroman) [60]. A study in rats also showed such activity for genistein [61]. The contribution of the gut microbiota is crucial in the bioavailability of isoflavones. Therefore, one way to improve bioavailability is to combine with cyclodextrins that increase bioavailability and have a prebiotic effect [62].

The digestive system is also important in the absorption of isoflavones. The absorption of isoflavones depends on their chemical form, including the presence of sugar residues in the form of glycosides. Isoflavones occur in plants as aglycones and glycosides. The glycosidic form can affect the bioavailability and absorption of isoflavones. In order for aglycones to exhibit biological activity, glycosides must be enzymatically hydrolyzed, releasing active aglycones. Enzymatic hydrolysis of the glycosides by intestinal beta-glucosidases is essential for the release of isoflavone aglycones [63].

### 2.8. Principal Component Analysis (PCA)

**Figure 8:**
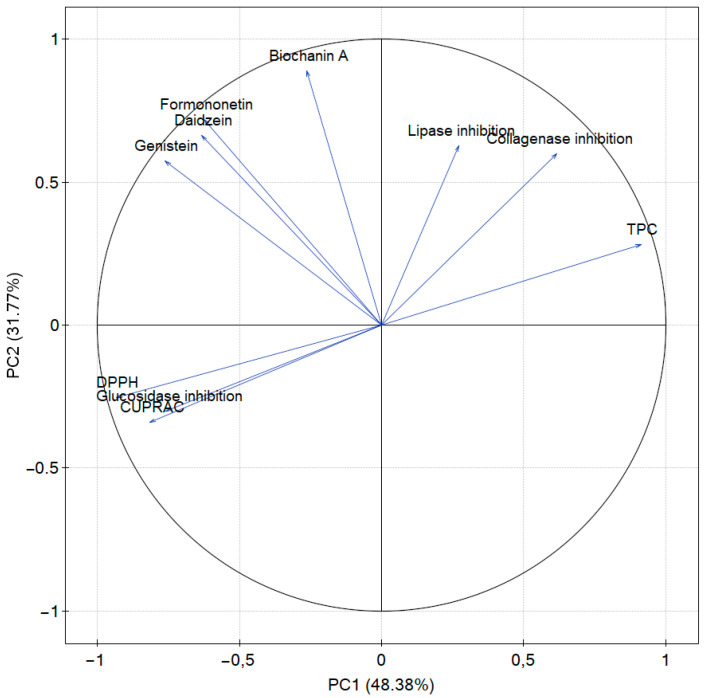
The relationship of red clover leaf and flower varieties on the factorial plane formed by the first two principal components, where individual varieties were denoted as leaves—‘Tenia’ (1), ‘Atlantis’ (2), ‘Lucrum’ (3), ‘Magellan’ (4), ‘Lemmon’ (5), ‘Milena’ (6); flowers—‘Tenia’ (7), ‘Atlantis’ (8), ‘Lucrum’ (9), ‘Magellan’ (10), ‘Lemmon’ (11), ‘Milena’ (12).

PCA was used to group the extracts according to their chemical composition, ability to inhibit enzymes and antioxidant activity. The first two principal components (PCs) explained 80.15% of the total variability, with 48.38% for PC1 and 31.77% for PC2. PC1 was positively correlated with total polyphenol content, lipase and collagenase inhibitory activity, and negatively correlated with isoflavones and antioxidant activity and glucosidase inhibition, whose values were expressed as IC_50_. PC2 was negatively correlated with IC_50_ values and positively correlated with total phenolic compounds, lipase and collagenase inhibitory capacity and isoflavones (Figure 8).

**Figure 9:**
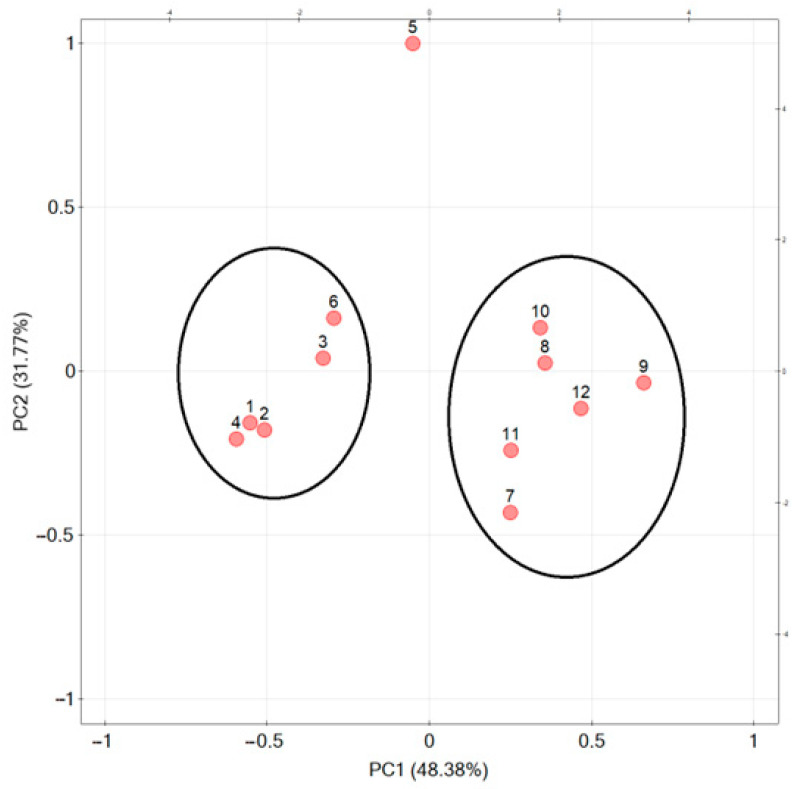
Principal component analysis (PCA) showing the factor loading plot.

Figure 9 shows the interrelationships of the positioning of the analyzed varieties (leaves and flowers) in comparison to others. Analyzed samples of the same part of the raw material were grouped together regardless of variety, indicating that the part of the raw material has a greater influence on the parameters analyzed than the variety. As described in the individual analyses before, the variety ‘Lemmon’ shows different characteristics, and assigning the variety to a group is impossible.

The analysis showed that isoflavone content is not correlated with antioxidant activity or enzyme inhibition. It may therefore be suspected that the isoflavones present in the raw material are not mainly responsible for the biological activity investigated.

## 3. Materials and Methods

### 3.1. Experimental Material

Plant material for the study of six red clover (*Trifolium pratense* L.) varieties came from experimental fields belonging to the Central Research Centre for Cultivar Testing in Słupia Wielka, near Poznań. The experimental field was located on light soil, class IIIa (pH 7.2). The content of basic nutrients was at the following level: P_2_O_5_ (22.1 mg/100 g of soil), K_2_O (16.0 mg/100 g of soil) Mg (4.4 mg/100 g of soil). During the growing season, red clover plants were fed with Lubofosf 12 granular fertilizer. Six varieties of raw materials were tested:

- Tenia—a diploid, medium-early variety from Małopolska Hodowla Roślin Spółka z o.o., entered in the National Register in 2006. Plants of this variety are characterized by very good winter hardiness. It has a very fast rate of plant regrowth in spring. After swathing, the rate of plant regrowth is rated as average. During the growth period, this variety shows a low tendency to lodge. It is distinguished by a significantly higher seed yield than other red clover varieties. Plants of this variety show high total protein content and high protein yield during the growing season.
- Milena—a diploid variety from Małopolska Hodowlia Roślin Spółka z o.o., entered in the National Register in 2008. It has good winter hardiness and a low tendency to lodge. The regrowth rate in the early spring vegetation period and after mowing is assessed as average. The variety shows good resistance to clover canker and powdery mildew infestation. Intended for hay use in open field cultivation. Performs well in pure sowing and in mixtures with grasses. Pure sowing gives a high yield of fresh and dry matter with very good quality parameters. Plants show a high content of total protein in dry matter. It yields fairly well when grown for seed and the seed yield ranges from 300 to 600 kg per ha.
- Atlantis—a medium-early tetraploid variety from DSV company. The variety is characterized by good winter hardiness. It scores highly in terms of dry matter yields in all offshoots. In addition to an even yield distribution over the growing season, it has the additional characteristic of being highly competitive in the sward, thanks to its high plant growth. It stays in the sward for quite a long time and also has medium resistance to disease infestation.
- Lucrum—a diploid variety. In the year of sowing, it has a medium tendency to flower. The plant is characterized by a stem of medium length. Plants show a tendency to flower late.
- Magellan—a tetraploid variety. In the year of sowing, it is characterized by a weak tendency to flower. It is characterized by a medium stem length, which is characterized by medium hairiness. Plants tend to flower moderately in the year of sowing.
- Lemmon—a diploid variety. It shows a medium tendency to flower in the year of sowing, a stem of medium length, sparse to medium stem hairiness, and medium tendency to flower in the year of sowing.

### 3.2. Extraction

The extracts were prepared with 500 mg of lyophilized fresh leaves and flowers. Extractions were carried out using an ultrasonic bath (Ultrasonix cleaner proclean 10.0S, Ulsonix, Zielona Góra, Poland) at 50 °C. The extrahent was a 50:50 (*v*/*v*) mix of ethanol and water. The extraction was carried out in triplicate by pouring 50 mL of solvent each time. The extracts were collected and evaporated on a rotary evaporator at 50 °C to the 10 mL of solution. Samples were stored at −20 °C. The final concentration was 50 mg/mL.

### 3.3. Determination of the Content of Active Compounds Using HPLC Method

The content of active compounds was assessed using an HPLC (high-performance liquid chromatography) system, with a PDA 100 detector (Dionex Thermoline Fisher Scientific, Waltham, MA, USA) with Chromeleon software version 7.0, and a LiChrospher RP18-5column (5 μm, 250 × 4.6 mm). The mobile phase consisted of acetic acid/methanol/water (1:10:89) (A) and 1% acetic acid in methanol (B), in a gradient: 0 min: 80%A, 20%B; 30 min 30%A, 70%B; 39–40 min: 90%A, 10%B; 40.5–50 min: 80%A, 20%B). The identification of the compounds was achieved by comparing their retention times and UV spectra with the corresponding standards. Quantitative analysis was performed by measuring the peak area, utilizing the appropriate standard curve. Validation parameters for each standard are presented in Table S1 of the Supplementary Materials. Chromatograms with retention times of the test compounds (genistein, daidzein, biochanin, formononetin) are presented in Figure S1 of the Supplementary Materials.

### 3.4. Total Phenolic Content (TPC)

The TPC was determined by the modified Folin–Ciocalteu method [13]. In summary, 25.0 µL of each extract or different concentrations of gallic acid solution were mixed with 200.0 µL of distilled water and 15.0 µL of Folin–Ciocalteu reagent. The mixture was allowed to react at room temperature for 3 min. Subsequently, 60.0 µL of a 20.0% (*w*/*v*) aqueous solution of sodium carbonate was added, and the mixture was incubated in 96-well plates at room temperature for 30 min. The absorbance was measured at 760 nm using a plate reader (Multiskan GO 1510, Thermo Fisher Scientific, Vantaa, Finland). The blank sample contained water instead of the extract or gallic acid solution. The results were expressed as gallic acid equivalents (GAE) in milligrams per gram of plant material.

### 3.5. Antioxidant Activity

#### 3.5.1. DPPH Assay

In summary, a 96-well plate was used for the assay. Each well contained 25.0 µL of the test sample and 175.0 µL of a DPPH radical solution (3.9 mg/50 mL) in methanol. The control sample consisted of the DPPH radical solution and the solvent used for extraction, while the blank represented the absorbance value obtained from combining 25.0 µL of the extraction solvent with 175.0 µL of the solvent used to dissolve the DPPH radical (methanol). This procedure was repeated six times. The plate was shaken for 5 min at 25 °C and then incubated for 25 min at room temperature. The absorbance was measured at 517 nm using a plate reader (Multiskan GO 1510, Thermo Fisher Scientific, Vantaa, Finland). The free radical scavenging capacity (%) of the test extracts and the standard substance was calculated using the following equation:

(1) $${DPPH}_{scavenging\;activity}\left(\%\right)=\frac{A_{0}-A_{1}}{A_{0}}\times 100\%$$

where *A*_0_ is the absorbance of the control, and *A*_1_ is the absorbance of the tested sample.

#### 3.5.2. CUPRAC Assay

To create the CUPRAC reagent, a conical flask wrapped in aluminum foil was used. Equal volumes of a 7.5 mM neocuproine solution in 96% ethanol, a 10 mM aqueous copper chloride solution and an ammonium acetate buffer solution (pH = 7) were added to the flask. In the 96-well plate, 50.0 µL of the test sample and 150.0 µL of the CUPRAC reagent solution were combined. The blank consisted of a mixture of the CUPRAC reagent and the solvent used for extraction. The assay was replicated six times. The plate was covered with aluminum foil, shaken for 5 min at 25 °C and then incubated at room temperature for 25 min. The absorbance was measured at 450 nm using a plate reader (Multiskan GO 1510, Thermo Fisher Scientific, Vantaa, Finland).

The test results were expressed as an IC_0_._5_ value, which represents the concentration of the test or standard substance at which the absorbance value is 0.5. This value was determined using a polynomial trend line.

### 3.6. α-Glucosidase Inhibitory Assay

In summary, in a 96-well plate, 50 μL of the sample solution (50 μg/mL), 50 μL of 0.1 M phosphate buffer (pH 6.8) and 30 μL of α-glucosidase solution (0.5 U/mL) were combined and preincubated at 37 °C for 15 min. Following this, 20 μL of a 5 mM p-nitrophenyl-α-D-glucopyranoside (pNPG) solution in 0.1 M phosphate buffer (pH 6.8) was added and incubated at 37 °C for 20 min. To stop the reaction, 100 μL of a 0.2 M sodium carbonate solution was added to the mixture. The absorbance of the released p-nitrophenol was measured at 405 nm using a plate reader (Multiskan GO 1510, Thermo Fisher Scientific, Vantaa, Finland). The absorbance of the sample without the extract was used as the control, while the absorbance in the absence of the enzyme served as the blank control. The positive control was a sample containing a solution of acarbose instead of the extract tested. The degree of enzyme inhibition, expressed as a percentage, was calculated using the following formula:

(2) $$Inhibitory\;activity\;(\%)=[(A_{0}\;–\;A_{1})/A_{0}]\times 100$$

where *A*_0_ is the absorbance of the control (100% enzyme activity), and *A*_1_ is the absorbance of the tested sample.

### 3.7. Collagenase Inhibition Assay [44]

The collagenase inhibitory activity of each sample was determined in vitro according to Widodo et al. with some modifications. In summary, 15.0 μL of enzyme, 60 μL of tricine buffer (pH 7.5) and 30 μL of extracts (50 mg/mL) were mixed and incubated at 37 °C for 20 min. Next, 20 μL of FALGPA (0.5 mM) was added to the mixture, and the absorbance was immediately measured at 325 nm using the plate reader (Multiskan GO 1510, Thermo Fisher Scientific, Vantaa, Finland). The absorbance was measured again after 20 min of incubation at 37 °C following the addition of the substrate. A control sample containing only the solvent used instead of the extract was performed, and the results were reduced by the value of the blank representing the enzyme-free sample. The positive control was a sample containing a solution of epigallocatechin gallate instead of the extract tested. The collagenase inhibition rate (presented for the final concentration of substance in enzymatic reaction) expressed as a percentage of inhibition was calculated using Formula (2).

### 3.8. Lipase Inhibition Assay

The lipase assay was carried out using the method described by Buchholz et al. [64] with minor modifications. Pancreatic lipase enzyme solution (10 mg/mL) from porcine pancreas (Sigma-Aldrich, Merck, St. Louis, MO, USA) was prepared in Tris buffer (pH = 8.0), centrifuged and stored at 2–8 °C until use. The substrate used in this study was 30 mg p-nitrophenyl palmitate (Sigma-Aldrich, Merck) dissolved in 10 mL isopropanol and made up to a volume of 100 mL with a warm mixture containing 100 mg sodium deoxycholate, 50 mg gum arabic and 2 mL Triton X. The reaction mixture (30 μL of pancreatic lipase, 100 μL of buffer (pH = 8.0), 20 μL of extracts (50 mg/mL)) was incubated at 37 °C for 20 min. Then, 130 μL of *p*-nitrophene palmitate solution was added and incubated again at 37 °C for 30 min and the absorbance (A) was measured at 405 nm (Multiskan GO 1510, Thermo Fisher Scientific, Vantaa, Finland). A control sample containing only the solvent used instead of the extract was performed, and the results were reduced by the value of the blank representing the enzyme-free sample. The positive control was a sample containing a solution of orlistat instead of the extract tested (Formula (2)).

### 3.9. Parallel Artificial Membrane Permeability Assay (PAMPA)

To evaluate the effective permeability (Pe) of the analyzed compounds, the PAMPA-BBB, PAMPA-GIT and PAMPA-SKIN methods were used (Pion Inc., Billerica, MA, USA). The stock solutions of examined compounds were prepared with extracts (50 mg/mL) and diluted with Prisma buffer (pH = 7.4; Prisma HT, Pion Inc., Billerica, USA) to obtain the donor solutions (12.5 mg/mL). Then, 200 μL of the donor solution was added to the donor wells. Subsequently, each filter membrane of the top plate was coated with a suitable lipid solution, i.e., 5 μL BBB, GIT or preincubated with hydration buffer 24 h earlier for SKIN assay (Pion Inc., Billerica, MA, USA), and the acceptor well was filled with 200 μL of acceptor fluid. The acceptor plate and the donor plate were coupled together and incubated for 4 h at 37 °C. After incubation, the plates were separated, and the concentrations of compounds that crossed the membrane were determined using HPLC. To calculate *P_app_*, the following equation was used:

(3) $$P_{app}=\frac{-ln\left(1-\frac{C_{A}}{C_{equilibrium}}\right)}{S\times \left(\frac{1}{V_{D}}+\frac{1}{V_{A}}\right)\times t}$$

where *P_app_*—effective permeability coefficient (cm/s), *V_D_*—donor volume, *V_A_*—acceptor volume, *C_equilibrium_*—equilibrium concentration, *S*—membrane area, and *t*—incubation time (in seconds).

Compounds with *P_app_* < 1 × 10^−6^ cm/s are classified as having low permeability and those with *P_app_* > 1 × 10^−6^ cm/s as highly permeable compounds. Samples were analyzed in triplicate.

### 3.10. Statistical Analysis

Statistical analysis was carried out using Statistica 13.3 software (StatSoft Poland, Krakow, Poland). The normality of data distribution was assessed using the Shapiro–Wilk test. Statistical significance was determined using a one-way analysis of variance (ANOVA) followed by the Tukey post hoc test. Results were considered significant at a *p*-value of less than 0.05. To analyze correlations, principal component analysis (PCA) was performed using PQStat Software v. 1.8.4.142 (2022).

## 4. Conclusions

Statistically significant differences between the six varieties of red clover leaves and flowers were observed in the context of antioxidant enzyme inhibition activity and the chemical composition of the raw material. The raw material was shown to have the ability to inhibit enzymes relevant to diabetes, obesity and collagenase, an enzyme crucial for connective tissue. The evaluation of the activity of the two parts of the plant—the leaves and flowers—showed that the leaves have a higher content of isoflavones (genistein, daidzein, formononetin and biochanin A), but the flowers have a higher total polyphenol content, which translates into their stronger biological activity in terms of the studies conducted. The next step in investigating the potential of red clover should be to determine which compounds are responsible for the antidiabetic, antioxidant, lipase-inhibitory, collagenase and glucosidase activities. According to the study, despite the higher content of isoflavones, the leaves show lower activity in these domains. It is suggested that a synergistic effect between the polyphenols present in the extract may be key here, or that other compounds in addition to the widely studied isoflavones are responsible for this activity. Continued research may be key to fully understanding the potential of red clover.

The ‘Lemmon’ variety showed interesting activity; specifically, the leaves of this variety are characterized by their high isoflavone content and high potential in in vitro studies. This variety may be key to the development of research into the activity of the raw material, as it has two advantages—a high content of isoflavones responsible for estrogenic activity and a high content of all polyphenols, and thus the high activity tested in this study.

Permeability studies have shown that biochanin A and formononetin, present in the raw material, are able to pass through biological membranes by passive diffusion, while this effect was not observed for genistein and biochanin. In conclusion, the significant differences in red clover varieties show how important the choice of plant material variety is not only for resistance to environmental conditions and other important aspects from a cultivation point of view, but can also have a great impact on the biological activity of the raw material. The choice of variety is crucial for the holistic health effects of the raw material.

## Figures and Tables

**Table 1:** Total phenolic content (TPC) in six varieties of red clover (*Trifolium pratense* L.) leaves and flowers.

Total Polyphenol Content (TPC)		
	Leaves	Flowers
	mg GAE/g DW	
Tenia	15.922 ± 1.321 ^a^	27.909 ± 1.624 ^b^
Atlantis	16.747 ± 0.547 ^ab^	42.303 ± 1.264 ^a^
Lucrum	18.397 ± 1.423 ^b^	56.178 ± 1.102 ^c^
Magellan	12.758 ± 0.263 ^d^	42.575 ± 1.106 ^a^
Lemmon	40.584 ± 0.976 ^e^	43.401 ± 1.317 ^a^
Milena	23.124 ± 0.650 ^c^	42.032 ± 1.004 ^a^

Data expressed as mean ± SD; means followed by a common letter are not significantly different by one-way ANOVA with Tukey’s post hoc test (*p* > 0.05).

**Table 2:** Antioxidant activity of six varieties of red clover (*Trifolium pratense* L.) leaves and flowers.

	CUPRAC		DPPH	
	Leaves	Flowers	Leaves	Flowers
	IC_50_ (mg/mL)		IC_50_ (mg/mL)	
Tenia	2.049 ± 0.108 ^a^	1.206 ± 0.012 ^a^	6.352 ± 0.262 ^a^	3.033 ± 0.129 ^a^
Atlantis	1.856 ± 0.062 ^b^	0.814 ± 0.0012 ^b^	6.469 ± 0.106 ^a^	2.280 ± 0.039 ^b^
Lucrum	1.710 ± 0.029 ^b^	0.644 ± 0.004 ^c^	4.854 ± 0.273 ^b^	1.393 ± 0.096 ^c^
Magellan	3.363 ± 0.067 ^c^	0.955 ± 0.019 ^d^	7.924 ± 0.082 ^c^	2.375 ± 0.060 ^d^
Lemmon	0.683 ± 0.0037 ^d^	0.832 ± 0.0017 ^b^	2.583 ± 0.089 ^d^	2.197 ± 0.057 ^be^
Milena	1.266 ± 0.045 ^e^	0.723 ± 0.029 ^e^	4.090 ± 0.029 ^e^	2.125 ± 0.044 ^bf^
Ascorbic acid	0.073 ± 0.003		0.068 ± 0.005	

Data expressed as mean ± SD; means followed by a common letter are not significantly different by one-way ANOVA with Tukey’s post hoc test (*p* > 0.05).

**Table 3:** Antidiabetic activity of six varieties of red clover (*Trifolium pratense* L.) leaves and flowers at a concentration of 50 mg/mL.

α-Glucosidase Inhibition		
	Leaves	Flowers
	IC_50_ (mg/mL)	
Tenia	13.674 ± 0.653 ^a^	5.357 ± 0.375 ^a^
Atlantis	24.935 ± 0.604 ^b^	4.903 ± 0.147 ^a^
Lucrum	15.417 ± 0.396 ^a^	2.044 ± 0.104 ^b^
Magellan	14.768 ± 0.263 ^a^	5.043 ± 0.156 ^a^
Lemmon	4.481 ± 0.263 ^d^	17.006 ± 0.918 ^c^
Milena	13.879 ± 1.083 ^a^	3.711 ± 0.074 ^a^
Acarbose	IC_50_ = 2.871 ± 0.044 mg/ml	

Data expressed as mean ± SD; means followed by a common letter are not significantly different by one-way ANOVA with Tukey’s post hoc test (*p* > 0.05).

**Table 4:** Anticollagenase activity of six varieties of red clover (*Trifolium pratense* L.) leaves and flowers at a concentration of 50 mg/mL.

Collagenase Inhibition		
	Leaves	Flowers
	Inhibition (%)	
Tenia	28.920 ± 4.473 ^a^	49.468 ± 4.639 ^a^
Atlantis	34.776 ± 3.010 ^ba^	72.188 ± 3.568 ^b^
Lucrum	41.625 ± 2.005 ^bc^	89.174 ± 4.206 ^c^
Magellan	46.041 ± 4.59 ^cf^	63.483 ± 3.330 ^c^
Lemmon	89.381 ± 4.780 ^d^	42.036 ± 3.206 ^a^
Milena	40.261 ± 3.780 ^b^	47.894 ± 2.526 ^a^
EGCG	IC_50_ = 0.666 ± 0.007 mg/ml	

Data expressed as mean ± SD; means followed by a common letter are not significantly different by one-way ANOVA with Tukey’s post hoc test (*p* > 0.05).

**Table 5:** Antilipase activity of six varieties of red clover (*Trifolium pratense* L.) leaves and flowers at a concentration of 50 mg/mL.

Lipase Inhibition		
	Leaves	Flowers
	Inhibition (%)	
Tenia	5.321 ± 1.281 ^a^	11.318 ± 1.888 ^a^
Atlantis	19.679 ± 3.758 ^b^	28.057 ± 1.357 ^b^
Lucrum	55.605 ± 5.602 ^c^	76.583 ± 4.525 ^b^
Magellan	33.699 ± 2.671 ^d^	66.301 ± 7.056 ^c^
Lemmon	72.044 ± 5.371 ^e^	36.402 ± 4.112 ^d^
Milena	20.861 ± 3.451 ^ba^	60.094 ± 3.774 ^c^
Orlistat	IC_50_ = 0.400 ± 0.026 mg/ml	

Data expressed as mean ± SD; means followed by a common letter are not significantly different by one-way ANOVA with Tukey’s post hoc test (*p* > 0.05).

## Data Availability

The data presented in this study are available from the authors upon reasonable request.

## Supplementary Materials

The following supporting information can be downloaded at: https://www.mdpi.com/article/10.3390/molecules28135178/s1, Table S1: Validation parameters for standard; Figure S1: Chromatograms with retention times of the test compounds (genistein, daidzein, biochanin, formononetin) and of the sample extract.

## Sample Availability

Samples of the compounds are available from the authors.
