# Supplementary material for: Multidirectional Effects of Red Clover (Trifolium pratense L.) in Support of Menopause Therapy
Source: Molecules. 2023 Jul 3;28(13):5178. doi: 10.3390/molecules28135178 (PMC10343205; doi:10.3390/molecules28135178)
Supplement: Supplementary file 1 [file molecules-28-05178-s001.zip › molecules-2472573-supplementary.pdf]

molecules

MDPI

Supplementary Materials

Table S1. Validation parameters for standard.

Parametr	Genistein	Daidzein	Formononetin	Biochanin A
Linearity: y = ax + b				
a ± S_a	698.76 ± 2.41	439.08 ± 7.71	486.29±9.92	650.13±31.82
b ± S_b	nonsignificant (α=0,05)	nonsignificant (α=0,05)	nonsignificant (α=0,05)	nonsignificant (α=0,05)
Correlation coefficient (r)	0,9999	0.99982	0.9975	0.9985
Linearity [mg/ml]	0.011 – 0.719	0.001-0.727	0.010-0.640	0.010-0.682
RSD (%)				
Low content	0.2138	0.4468	4.3483	1,3263
Medium content	0.7391	0.4791	2.8847	0,5313
High content	0.6603	0.1145	2.2072	0,5865
Limit of detection (LOD) [µg/ml]	0.0025	0.0164	0.0133	0.0341
Limit of quantification (LOQ) [µg/ml]	0.0077	0.0496	0.0403	0.1034

S_a - standard deviation of the slope; S_b - standard deviation of the intersection point. t. calculated values of the Student's t-test. $t\alpha.f$ = 3.182 Critical values of the Student's test for degrees of freedom f = 3 and significance level α = 0.05.

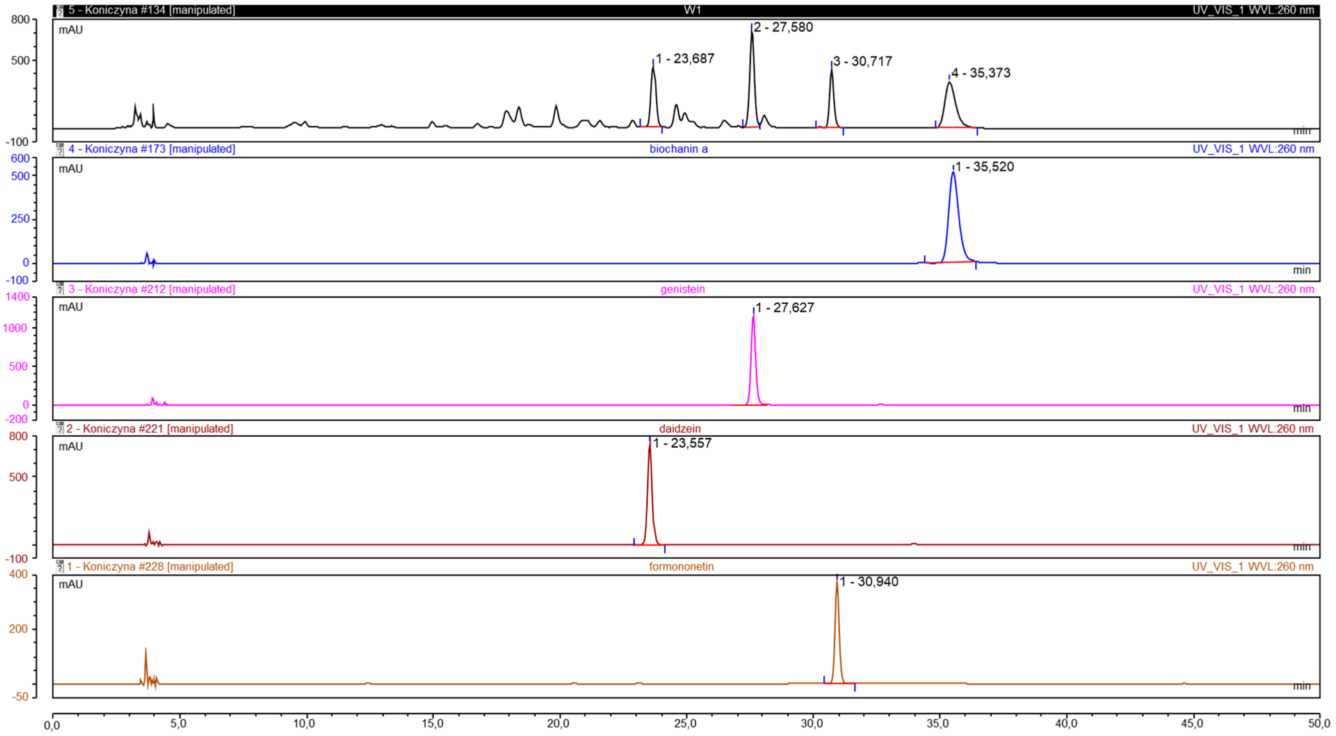

Figure S1. Chromatograms with retention times of the test compounds (genisten, daidzein, biochanin, formononetin) and of the sample extract.
